# Prognostic value of the obstetric specific scoring systems and four years’ experience of a tertiary center

**DOI:** 10.1186/s12884-025-08457-4

**Published:** 2025-12-24

**Authors:** Esra Karatas, Osman Onur Ozkavak, Nazlı Orhan, Betul Akgun Aktas, Namık Nebi Ozcan, Atakan Tanacan, Ozgur Kara, Dilek Sahin

**Affiliations:** 1 Department of Obstetrics and Gynecology, Ministry of Health, Ankara Bilkent City Hospital, Ankara, 06800 Turkey; 2 Department of Anesthesiology and Reanimation, Ministry of Health, Ankara Bilkent City Hospital, Ankara, Turkey

**Keywords:** Maternal sepsis, Obstetric sepsis, Perinatal outcome, Septic shock

## Abstract

**Background:**

Obstetric sepsis is a significant and preventable cause of maternal mortality. The obstetric modified SOFA (omSOFA) score was created to diagnose sepsis in this population, and the sepsis in obstetrics score (SOS) was developed to predict the need for ICU care. This study investigated the prognostic value of these scores in patients treated in the ICU for obstetric sepsis, analyzed the patients’ characteristics, and examined the perinatal outcomes associated with sepsis.

**Methods:**

The study was retrospectively designed, and patients hospitalized in the ICU due to maternal sepsis were evaluated. These patients were divided into two groups based on the length of ICU stay. An ICU stay exceeding 72 h is considered a prolonged stay. The data, including demographics, laboratory results and obstetric outcomes, were compared between the groups. Receiver operating characteristic (ROC) curve analyses were performed to investigate the performance of omSOFA and SOS scores in predicting prolonged ICU stay and septic shock.

**Results:**

The study included 25 women with maternal sepsis. Sepsis developed during pregnancy in 40% of patients, after cesarean section in 28%, after abortion in 20%, and after vaginal delivery in 12%. Septic shock developed in 12% (*n* = 3) of patients during ICU hospitalization. SOS showed better performance in predicting the need for ICU longer than 72 h (cut-off: 3, AUC:0.865, CI:0.721-1.000, sensitivity:75%, specificity:100% PPV:100% NPV:92.7% *p* = 0.003). At the same time, omSOFA performed better in predicting septic shock (cut-off: 5, AUC:0.985, CI: 0.941-1.000, sensitivity:100% specificity:95%, PPV:73.2%, NPV:100% *p* = 0.007).

**Discussion:**

Considering that prolonged stay in the ICU and development of septic shock are important determinants of morbidity and mortality, SOS and omSOFA scores could serve as valuable prognostic tools in obstetric sepsis cases.

## Introduction

Sepsis is recognized as a significant global health issue. The definition of the condition is as follows: “life-threatening organ dysfunction caused by an irregular host response to infection” [1]. Obstetric sepsis is defined as the occurrence of the aforementioned condition during the pregnancy and the postpartum period [2]. The global maternal mortality rate in 2020 was 223 per 100,000 live births (for 185 countries and territories). Developing countries are disproportionately responsible for this rate [3]. The maternal mortality rate resulting from sepsis is approximately 6.6%, with the majority of these deaths occurring in the postpartum period [4]. Even though sepsis appears to be responsible for a relatively limited proportion of maternal mortality, it is a largely preventable cause of maternal death, provided that it is diagnosed and treated promptly [4].

To reduce morbidity and mortality in cases of sepsis, various scoring systems have been developed to identify patients at risk of clinical deterioration during the early stages [5–7]. The Quick Sequential Organ Failure Assessment (qSOFA) is the most widely used tool globally for identifying patients at risk of developing sepsis in cases of infection [8, 9]. The SOFA scoring system is widely used to assess the severity of organ dysfunction and to diagnose sepsis in patients identified as high risk by qSOFA. These scoring systems are useful and widely used tools in the non-pregnant adult population. However, they are insufficient for predicting and diagnosing sepsis in pregnant and postpartum patients. This may be because physiologic changes during pregnancy and the postpartum period are not considered [10, 11]. Therefore, using scoring systems that consider the physiological effects of pregnancy has been suggested for the diagnosis and prediction of sepsis in obstetric patients. The main ones are the Sepsis in Obstetrics score (SOS) proposed by the Society for Maternal-Fetal Medicine (SMFM) and the American College of Obstetrics and Gynecology (ACOG), and the modified obstetric SOFA (omSOFA) created by the Society of Obstetric Medicine Australia and New Zealand (SOMANZ) [12–15]. Despite the clear benefits of these scoring systems for clinical use, they have not yet achieved widespread implementation [16, 17].

Numerous factors contribute to determining the prognosis in cases of sepsis. These factors include age, comorbidities, degree of organ dysfunction, serum lactate level, and length of hospitalization [18–21]. It has been documented that an intensive care unit stay lasting more than 72 h is indicative of severe sepsis rather than uncomplicated infections [6]. Septic shock represents the terminal phase of the sepsis spectrum, with a high mortality rate if not identified promptly and managed appropriately.

The primary objective of the present study is to evaluate the predictive value of scoring systems developed for the diagnosis and management of sepsis in the obstetric population, specifically in predicting prolonged hospitalization and the development of septic shock, which are important prognostic factors. The secondary objective is to share the experience of our tertiary referral center, which receives critically ill obstetric patients from all over the country.

## Methods

### Study design

The study was designed as a retrospective cohort study. Patient data were obtained from the hospital’s electronic record system and patient files. All investigators adhered to the principles outlined in the Declaration of Helsinki. The study was approved by the institutional ethics committee prior to the initiation of the research (approval number: 1–24-713). The Institutional Ethics Committee decided that informed consent was not required due to the retrospective nature of the study. The patient sample selected for inclusion in the study comprised individuals diagnosed with sepsis during pregnancy or in the puerperal period (42 days after delivery of the placenta) and subsequently treated in the intensive care unit (ICU) between February 2021 and February 2025. The study was conducted at a perinatology center with a Level III intensive care unit. High-risk obstetric cases in our ICU are managed in a multidisciplinary manner under the joint supervision of anesthesia and perinatology specialists.

The inclusion criteria were a diagnosis of obstetric sepsis and treatment in an intensive care unit. The study excluded patients who were pregnant or postpartum and had been admitted to the intensive care unit for another condition, patients with infections requiring hospitalization but not diagnosed with sepsis and those who did not require intensive care. The diagnosis of sepsis was made in accordance with the SOFA criteria, as recommended by the ACOG and SMFM [14, 15]. As indicated in the aforementioned guidelines, septic shock was diagnosed in cases necessitating vasopressor treatment for a mean arterial pressure (MAP) of ≥ 65 mmHg and a serum lactate level of >2 mmol/L, despite adequate volume resuscitation. Only data from patients whose ICU and hospitalization ended at the start of the study were included in the analyses. The criteria for patient discharge from the intensive care unit included regression of the SOFA score during their hospitalization and improvement in organ dysfunction.

The vital signs (body temperature, pulse rate, systolic and diastolic blood pressure values, and respiratory rate) and the results of laboratory tests taken during the initial admission to the ICU were analyzed. The laboratory data that were studied included hemoglobin, white blood cell count (WBC), platelet count (PLT), aspartate aminotransferase (AST), alanine aminotransferase (ALT), total bilirubin, albumin, creatinine, procalcitonin, C-reactive protein (CRP), pH, and lactate values obtained by blood gas analysis. Furthermore, an evaluation was conducted to ascertain the correlation between the lowest Glasgow Coma Scale (GCS) scores calculated during ICU hospitalization and the type and duration of antibiotics used [22]. In addition, the results of blood, urine, and cervical culture and antibiogram tests were reviewed. The gestational age at diagnosis, obstetric and demographic data, duration of ICU stay, and the outcome of the pregnancy were also analyzed. The SOS and omSOFA scores were calculated based on vital parameters, laboratory values and examination findings recorded at the time of admission to the intensive care unit (Tables 1 and 2). Gestational age at birth, neonatal weight, first and fifth-minute APGAR scores, and the presence of NICU hospitalization were evaluated from neonatal outcomes.

**Table 1:** Components of sepsis in obstetric score (SOS)

Variable	High Abnormal Range				Normal	Low Abnormal Range			
	+ 4	+ 3	+ 2	+ 1	0	+ 1	+ 2	+ 3	+ 4
Temperature ( °C)	> 40.9	39-40.9		38.5–38.9	36-38.4	34-35.9	32-33.9	30-31.9	< 30
SBP (mmHg)					> 90		70–90		< 70
HR (beats per minute)	> 179	150–179	130–149	120–129	≤ 119				
RR (breaths per minute)	> 49	35–49		25–34	12–24	10–11	6–9		≤ 5
SpO2 (%)					≥ 92%	90–91%		85–89%	≤ 85%
WBC (µg/L)	> 39.9		25-39.9	17-24.9	5.7–16.9	3-5.6	1-2.9		< 1
% immature neutrophils			≥ 10		< 10				
Lactic acid (mmol/L)			≥ 4		< 4				

*SBP *systolic blood pressure,* HR *heart rate,* RR *respiratory rate,* SpO2 *blood oxygen saturation,* WBC *white blood cell count

**Table 2:** Components of obstetrically modified SOFA score

Parameter	Score		
	0	1	2
*Respiration*			
PaO2/FiO2 (mmHg)	≥ 400	300 to < 400	< 300
*Coagulation*			
Platelet (×10^9^/L)	≥ 150	100–150	< 100
*Liver*			
Bilirubin (µmol/L)	≤ 20	20–32	> 32
*Cardiovascular*			
Mean arterial pressure (mmHg)	MAP ≥ 70	MAP < 70	Vasopressors required
*Central nervous system*	Alert	Rousable by voice	Rousable by pain
*Renal*			
Creatinine (µmol/L)	≤ 90	90–120	> 120

*SOFA *Sequential (sepsis-related) Organ Failure Assessment score,* FIO*_*2*_ fraction of inspired oxygen (expressed as a decimal),* PaO*_*2*_ partial pressure of oxygen (in mmHg),* MAP *mean arterial pressure mmHg

Given the documented findings in previous studies that ICU length of stay exceeding 72 h is a poor prognostic factor, hospitalizations lasting beyond this duration were classified as prolonged ICU stay [23, 24]. Patients were divided into two groups: those with prolonged ICU stays and those without. A comparative analysis was conducted on the clinical, demographic, and laboratory data, as well as the SOS and omSOFA scores, between the two groups.

### Statistical analysis

The data were analyzed using the Statistical Package for the Social Sciences (SPSS 30, IBM SPSS Statistics for Windows, Version 30.0). Armonk, NY: IBM Corp.). The Shapiro-Wilk test was employed to assess the normality of the data distribution. Given the non-normal distribution of the data, the Mann-Whitney U test was employed to analyze the continuous variables. The results of this analysis are presented as median and interquartile range (IQR). Categorical variables were analyzed using the Pearson Chi-square test, and the results are presented as numbers and percentages. Receiver operating characteristic (ROC) curve analysis was performed to evaluate the performance of the omSOFA and SOS scores in predicting prolonged ICU stays (> 72 h) and the development of septic shock. Optimal cut-off values were determined by employing Youden’s index, and the sensitivity and specificity values were subsequently calculated. The values obtained were then used to calculate the positive and negative predictive values (PPV, NPV). A p-value less than 0.05 was considered statistically significant.

## Results

During the period included in the study, the hospital averaged 15,000 deliveries per year.

The study comprised a total of 25 patients with sepsis. Patients with an ICU stay of 72 h or less were included in group 1 (*n* = 9), and patients with an ICU stay of more than 72 h were included in group 2 (*n* = 16). The statistical analysis was based on these two groups.

There was no significant difference between the two groups in maternal age and nulliparity. A comparative analysis of the vital parameters of pregnant women in both groups showed no significant differences. Among the laboratory parameters, only the platelet count was significantly lower in group 2 (*p* = 0.047). No significant differences were found between the groups in terms of other hemogram parameters and inflammatory markers (CRP, procalcitonin). In addition, the median GCS score of group 2 was significantly lower than that of group 1 (*p* = 0.041). The demographic and clinical characteristics, vital parameters, and laboratory values of patients in both groups are summarized in Table 3.

**Table 3:** Comparison of demographic, laboratory, and clinical characteristics between groups

Variable	Group 1 (*n*:9)	Group 2 (*n*:16)	*p*-value
Age (year)	28.6 (8)	30.0 (15)	0.476^a^
Nulliparity	% 55.6 (5)	% 25 (4)	0.127^b^
Body temperature (°C)	37.36 (1.5)	37.70 (2.2)	0.513^a^
SBP (mmHg)	112.4 (9)	101.5 (22)	0.051^a^
DBP (mmHg)	60.5 (18)	55.1 (24)	0.223^a^
MAP (mmHg)	77.83 (16.97)	70.60 (23.83)	0.119^a^
Heart Rate (beats per minute)	96.6 (32)	125.4 (30)	0.15^a^
Respiratory Rate (breaths per minute)	21.7 (6)	23.1 (9)	0.475^a^
Hemoglobin (g/dL)	9.8 (1.9)	9.2 (1.7)	0.192^a^
WBC (×10^9^/L)	15366.67 (10655)	13672.50 (13300)	0.610^a^
PLT (×10^9^/L)	343.89 (310)	205.56 (214)	**0.047** ^**a**^
Total Bilirubin (mg/dL)	0.517 (0.33)	1.360 (0.56)	0.164^a^
AST (U/L)	29.0 (14)	181.06 (88)	0.156^a^
ALT (U/L)	20.56 (13)	80.81 (37)	0.821^a^
Creatinine (mg/dL)	0.56 (0.3)	0.97 (1)	0.350^a^
Albumin (g/L)	29.78 (4)	29.0 (8)	0.321^a^
GFR (ml/min/1.73m^2^)	130.11 (22)	102.88 (94)	0.308^a^
pH	7.43 (0.07)	7.37 (0.109)	0.257^a^
Lactate (mmol/L)	1.54 (1.18)	2.22 (1.60)	0.193^a^
Procalcitonin (µg/L)	15.48 (18.55)	59.07 (52.30)	0.396^a^
CRP (mg/L)	247.42 (175.85)	175.90 (174.68)	0.777^a^
Glasgow Coma Scale	15 (0)	14 (2)	**0.041** ^**a**^
Length of hospital stay (day)	10 (4)	15.5 (11)	**0.043** ^**a**^
Length of stay in ICU (day)	2.56 (1)	7.50 (4)	**< 0.001** ^**a**^

^*a*^ *Mann Whitney U test; results are presented as median (interquartile range)*

^*b*^ *Pearson chi square test; results are presented as percentage (number)*

*SBP *systolic blood pressure,* DBP *diastolic blood pressure,* MAP *mean arterial pressure mmHg,* WBC *white blood cell count,* PLT *platelet count,* AST *aspartate aminotransferase,* ALT *alanine aminotransferase,* GFR *glomerular filtration rate,* CRP *C-reactive protein,* ICU *The Intensive Care Unit

* p < 0.05 accepted as statistically significant*

A comprehensive analysis of the etiology of sepsis was performed, encompassing all cases included in the study, it was found that 44% of cases were due to urosepsis, 20% to septic abortion, 12% to pneumonia, 8% to endometritis, 8% to intra-abdominal abscess, 4% to surgical site infection and 4% to undetermined causes. The case of undetermined sepsis pertained to a 22-week pregnant patient who presented to the emergency department with severe joint pain and poor general condition. A preliminary diagnosis indicated that the patient’s symptoms were consistent with those associated with Crimean-Congo Hemorrhagic Fever (CCHF). However, the diagnostic accuracy of the tests performed could not be confirmed. The patient’s clinical condition improved with broad-spectrum antibiotherapy and supportive treatment, leading to the patient’s discharge. A statistically significant difference in the etiology of sepsis between the groups was not observed (*p* = 0.592) (see Table 4). Additionally, no significant difference was found in culture positivity rates between the groups (*p* = 0.361).

**Table 4:** Comparison of sepsis etiology and culture positivity between groups

Cause of Sepsis	Group 1 (9)	Group 2 (16)	Total (25)	*P* value^a^
Urosepsis	% 55.6 (5)	% 37.5 (6)	% 44 (11)	0.592
Septic abortion	% 11.1 (1)	% 25 (4)	% 20 (5)	
Pneumonia	% 11.1 (1)	% 12.5 (2)	% 12 (3)	
Endometritis	% 0 (0)	% 12.5 (2)	% 8 (2)	
Intraabdominal Abscess	% 11.1 (1)	% 6.3 (1)	% 8 (2)	
Undetermined (e.g., CCHF)	% 0 (0)	% 6.3 (1)	% 4 (1)	
Surgical site infection	% 11.1 (1)	% 0 (0)	% 4 (1)	
Culture positivity	% 66.7 (6)	% 18.3 (3)	% 36 (9)	0.361

^a ^*Pearson chi square test*,* results are presented as percentage (number)*

*CCHF *Crimean-Congo Hemorrhagic Fever

*p* *< 0.05 accepted as statistically significant*

Patients were divided according to the period in which they were diagnosed with sepsis: pregnancy, post-cesarean section, post-vaginal delivery, and post-abortion. Of the patients, 40% developed sepsis during pregnancy, 28% following a cesarean section, 20% after an abortion and 12% after vaginal delivery. A comparison of the periods of sepsis development between the two groups yielded no significant differences (Table 5).

**Table 5:** Comparation of pregnancy and postpartum status at the time of sepsis diagnosis

	Group 1 (9)	Group 2 (16)	Total (25)	*P* value^a^
Pregnancy	44% (4)	37.5% (6)	40% (10)	0.855
Post-cesarean section	33% (3)	25% (4)	28% (7)	
Post-abortion	11% (1)	25% (4)	20% (5)	
Post-vaginal delivery	11% (1)	12.5% (2)	12% (3)	

^*a*^ *Pearson chi square test*,* results are presented as percentage (number)*

*p* *< 0.05 accepted as statistically significant*

A total of 10 patients were admitted to the ICU due to sepsis during pregnancy. The pregnancy of one patient, at eight weeks of gestation, resulted in a spontaneous abortion following admission to the ICU. When the remaining nine pregnant patients were evaluated between the two groups, no significant difference were observed in terms of gestational week at the time of sepsis, birth week, neonatal weight, cesarean section rate, or neonatal intensive care unit admission. The first- and fifth-minute APGAR scores were significantly lower in group 2 compared to group 1 (*p* = 0.045, *p* = 0.009, respectively). The mean gestational age at the time of ICU admission for sepsis was 24.9 weeks, and the mean gestational age at delivery was 33.5 weeks. While no cases of intrauterine fetal death were detected in any patients, one preterm infant in group 2 died at an early neonatal period (Table 6).

**Table 6:** Comparison of obstetric and neonatal characteristics between the groups

Variable	Group 1 (*n*:3)	Group 2 (*n*:6)	*p*-value
Gestational age at time of sepsis (week)	25.6 (0)	27.3 (3.3)	0.516^a^
Gestational age at birth (week)	39 (0)	35 (7.0)	0.588^a^
Birth weight (gram)	3236 (0)	2306 (845)	0.302^a^
Cesarean rate	%66.6 (2)	%50 (3)	0.570^b^
1 st minute APGAR score	8 (0)	7 (1)	**0.045** ^a^
5th minute APGAR score	9 (0)	8 (1)	**0.009** ^a^
NICU admission	%33.3 (1)	%50 (3)	0.635^b^
Neonatal death	0	%16.7(1)	0.453^b^

^*a*^ *Mann Whitney U test; results are presented as median (interquartile range)*

^*b*^ *Pearson chi square test; results are presented as percentage (number)*

*NICU *neonatal intensive care unit

*p* *< 0.05 accepted as statistically significant*

A comparison of the groups in terms of SOS and omSOFA scores at the time of ICU admission revealed that both scores were significantly higher in the group with an ICU length of stay greater than 72 h (*p* = 0.003 and *p* = 0.004, respectively) (see Table 7). ROC curve analyses revealed that higher levels of both scores were statistically significantly predictive of the need for prolonged ICU stay. The cut-off values calculated to obtain optimal sensitivity and specificity values were 3 for SOS (sensitivity: 75%, specificity: 100%, PPV: 100%, NPV: 92.7%) and 1 for omSOFA (sensitivity: 81%, specificity: 78%, PPV: 53.8%, NPV: 92.9%).

**Table 7:** Comparison of SOS and OmSOFA scores between groups

Variable	Group 1 (*n*:9)	Group 2 (*n*:16)	*p*-value
**SOS**	0 (3)	5.5 (6)	**0.003** ^**a**^
**omSOFA**	0 (1)	2 (5)	**0.004** ^**a**^

^*a*^ *Mann Whitney U test*,* results are presented as median (interquartile range)*

*SOS* Sepsis in Obstetrics Score, *omSOFA*modified obstetric Sequential Organ Failure Assessment Score

*p < 0.05 accepted as statistically significant*

No maternal deaths were reported in the cohort. Septic shock developed in 12% (*n* = 3) of patients during their stay in the ICU. All patients who developed septic shock were in group 2. The ROC curve analyses conducted to examine the performance of the investigated scoring systems in predicting septic shock demonstrated that both scores exhibited statistical significance (*p* = 0.012 and *p* = 0.007, respectively). The sensitivity for both scores was 100%, while the specificity values were 92% for SOS and 95% for omSOFA. The NPV was found to be 100% for both scoring systems, with a PPV of 43.1% for SOS and 73.2% for omSOFA. In both the SOS and omSOFA scoring systems, the cutoffs for predicting septic shock were higher than those for predicting the need for a prolonged ICU stay (6 for SOS, 5 for omSOFA). The results of the ROC curve analysis and the prognostic performance values derived from this analysis are presented in Table 8.

**Table 8FV:** ROC curve analysis of SOS and omSOFA scores for predicting prolonged ICU stay and septic shock

Variable	AUC	Std. Error	95% Confidence Interval	Cutoff value	Sensitivity/Specificity	PPV/NPV	J value	*p* value
Prolonged ICU stay								
**SOS**	0.865	0.073	0.721-1.000	3	75%/100%	100%/92.7%	0.75	**0.003**
**omSOFA**	0.837	0.081	0.678–0.996	1	81%/78%	53.8%/92.9%	0.59	**0.006**
**Septic shock**								
**SOS**	0.955	0.049	0.859-1.000	6	100%/92%	43.1%/100%	0.909	**0.012**
**omSOFA**	0.985	0.022	0.941-1.000	5	100%/95%	73.2%/100%	0.955	**0.007**

*SOS *Sepsis in Obstetrics Score,* omSOFA *modified obstetric Sequential Organ Failure Assessment Score,* J value *The Youden Index

*p < 0.05 accepted as statistically significant*

## Discussion

Prolonged ICU hospitalization and the development of septic shock are significant poor prognostic factors in sepsis cases. To the best of our knowledge, this study is the first to evaluate the prognostic value of scoring systems developed to predict sepsis diagnosis and intensive care unit admission in the obstetric population. The findings indicate that these scores may also serve as valuable prognostic tools for patients hospitalized in the ICU for sepsis.

Sepsis is not classified as a specific disease but rather as a syndrome caused by an unclear pathophysiology [12]. Sepsis is defined as the development of life-threatening organ dysfunction as a result of the patient’s inappropriate immune response to infection [25]. In adult populations, the SOFA score is a reliable and objective tool for assessing organ failure. However, the physiologic changes observed during pregnancy limit the applicability of the SOFA score in the obstetric population. The existing publications on this subject are generally retrospective and report results from studies with small patient numbers [1, 15–17, 26].

Sepsis in obstetric patients can be quickly fatal. Therefore, immediate initiation of treatment and resuscitation is essential [12, 15, 26]. Research indicates that 63% of maternal sepsis-related deaths are attributable to inadequate healthcare, delays in diagnosis and management. Especially in the absence of maternal fever, sepsis diagnosis is often delayed [12, 26, 27]. Pregnancy is characterized by increased plasma volume, elevated cardiac output, and peripheral vasodilation. At the same time, normal laboratory values in obstetric patients differ from those observed in non-obstetric patients. For example, the values classified as leukocytosis in adult patients are considered normal in pregnant and postpartum patients. Similarly, a physiologically increased respiratory rate during pregnancy may be a sign of sepsis in non-pregnant patients. None of the existing definitions of sepsis considers these physiological changes of normal pregnancy. The physiological adaptations that occur during pregnancy, when combined with the high rates of trauma and surgical intervention that occur in the peripartum period, result in patients during pregnancy and the puerperium being at risk of developing infections that remain unrecognized until there is a significant clinical deterioration. This situation poses considerable challenges in the recognition of obstetric patients complicated by sepsis or septic shock.

Based on this, new scoring systems have been developed specifically for the obstetric population. The main ones are the Sepsis in Obstetrics (SOS) score and the obstetric modified SOFA (omSOFA) proposed by SOMANZ [12, 13]. The SOS score was developed to predict the need for ICU admission in obstetric patients, and the omSOFA score was designed to diagnose sepsis in obstetric patients. In a validation study of the SOS, the score demonstrated a sensitivity of 64%, a specificity of 88%, a PPV of 15%, and an NPV of 98.6% for ICU admission when the cut-off value was 6 [13, 16]. To our knowledge, there are no documented prospective validation studies for omSOFA. However, the available literature provides insufficient data regarding the predictive value of these scores for poor prognosis. The study identified prolonged stay in the ICU and development of septic shock as poor prognostic factors in patients requiring ICU admission for sepsis. It has been established that there is a positive correlation between prolonged stay in intensive care during pregnancy and the postpartum period and elevated morbidity and mortality rates [28, 29]. While SOS and omSOFA demonstrated comparable performance in terms of sensitivity and NPV in analyses predicting prolonged ICU stay, SOS appeared to exhibit superiority in specificity and PPV. This may be attributable to the objective parameters employed in the calculation of SOS, which utilizes a more extensive scoring scale, thereby enhancing its sensitivity and accuracy. Septic shock was observed in 12% of the patients included in the study. A subsequent analysis of the scores’ performance in predicting septic shock revealed that although there were minor discrepancies between the two scores in sensitivity, specificity, and negative predictive value, the positive predictive value of the omSOFA score was considerably higher than that of SOS. This finding can be attributed to the inclusion of a relatively invasive component in the omSOFA score, such as the PaO2/FiO2 ratio, as well as to the evaluation of parameters like MAP and vasopressor requirement. These components are also employed in the diagnosis of septic shock.

According to the extant literature, the rate of progression from sepsis to septic shock is approximately 15.8% in the non-pregnant population [30], while variable rates of 14–20% have been reported in the obstetric population [31, 32]. The hospital where the study was conducted is a tertiary care facility that receives patients from across the country. It has a specialized intensive care unit and team for obstetric patients, and it employs infectious disease specialists who exclusively treat obstetric patients. It is concluded that delays in diagnosis and treatment were less common in the current patient population, resulting in a lower incidence of septic shock compared to other studies.

According to recent data, the incidence of sepsis in pregnant patients is reported to be 0.001%, while in postpartum patients it ranges from 0.002% to 0.01%. The risk of developing postpartum sepsis is thought to be 2–10 times higher than in pregnant women [33–35]. In this cohort, 40% of the patients were pregnant (Table 6). The analysis of the etiology of sepsis in pregnant and postpartum patients reveals that pelvic causes are more prevalent in postpartum patients, while non-pelvic causes are more common in pregnant women [35, 36]. Among the causes of sepsis, the most common causes in the antepartum period are septic abortion, chorioamnionitis, urinary tract infections, and appendicitis, while the most common causes in the postpartum period are endometritis, wound infections, urinary tract infections, and pneumonia [26]. In this analysis, the most prevalent cause was urosepsis (44%), followed by septic abortion (20%) in the overall cohort (Table 5). Among pregnant women, urosepsis was the most common etiology (80%), and after vaginal delivery, endometritis accounted for the majority of cases (66.6%).

It is acknowledged that culture may not always be positive in cases of sepsis, and the source of infection cannot always be identified [27, 31]. Research has shown that microorganisms can be successfully cultivated in about two-thirds of the cultures collected to identify the causative agent in sepsis cases [16]. Despite the higher rate of positive blood cultures observed in patients with a higher risk of ICU admission in the SOS study, no statistically significant difference in culture positivity was observed between the two groups in this study. The most commonly detected microorganism in culture-positive patients was Escherichia coli, with a prevalence of 50%. The most prevalent microorganisms identified in the existing literature include E. coli and group A and B streptococci, although mixed infections are also observed [26, 31, 35]. The results of the isolated microorganisms are consistent with the extant literature.

It is a known fact that sepsis is a significant cause of both maternal and perinatal adverse outcomes. Pregnancy outcomes complicated with sepsis include miscarriage, preterm labor, low birth weight, fetal distress, intrauterine death, and neonatal death [37–39]. In 20% of the patients included in the study, there was a hospitalization in the ICU after an abortion. First trimester sepsis cases, particularly those precipitated by genital tract infection, almost always result in miscarriage [35]. In pregnancies resulting in a live birth, the mean time between admission to the ICU due to sepsis and delivery was calculated as 8.6 weeks. In this patient group, the most notable complication in the prenatal period was preterm labor (16.8%), while neonatal outcomes revealed neonatal death in one patient and neonatal ICU admission in 44.4%. In the group with prolonged ICU stay, gestational age at delivery and birth weight were lower, and the rate of neonatal ICU stay was higher. However, this difference was not statistically significant. Neonatal death occurred in the infant of a patient in the group with a prolonged ICU stay. Although the difference was not statistically significant, the increased rate of adverse perinatal outcomes in this group may be an indirect indicator of a severe systemic disorder that could compromise uteroplacental perfusion and fetal oxygenation. The increased rate of adverse outcomes highlights that sepsis is not only a maternal emergency but also poses serious risks to the fetus and newborn. Furthermore, these results suggest that prolonged ICU hospitalization for sepsis in pregnant women may be associated with poor perinatal outcomes, even after discharge. However, further studies containing more patient data are required to reach an accurate conclusion on this matter.

The limitations of this study include its retrospective design, its single-center setting, and its relatively small sample size. However, it is important to note that cases of obstetric sepsis requiring intensive care follow-up are not commonly encountered. The strengths of the study include its evaluation of the performance of the SOS and omSOFA scores—developed to assess the diagnosis of sepsis and the need for ICU admission in the obstetric population—in predicting adverse outcomes such as prolonged ICU stay and septic shock, as well as its contribution to the literature regarding the clinical characteristics and perinatal outcomes of obstetric sepsis cases.

In conclusion, prolonged ICU stay and the development of septic shock are significant determinants of poor prognosis in obstetric sepsis. The SOS and omSOFA scores demonstrated promising predictive performance for these adverse outcomes. These tools have the potential to serve as practical instruments to facilitate early risk stratification and timely intervention in critically ill obstetric patients. The integration of these scoring systems into standard evaluation protocols has the potential to standardize the assessment of disease severity and enhance maternal and perinatal outcomes. However, larger, multicenter, prospective studies are required to validate these results and establish the prognostic utility of these scores in clinical practice.

## Data Availability

The data that support the findings of this study are available on request from the corresponding author.
